# *N*′-(2,4-Dinitro­phen­yl)acetohydrazide

**DOI:** 10.1107/S1600536808019685

**Published:** 2008-07-09

**Authors:** Muhammad Zia-ur-Rehman, Mark R. J. Elsegood, Shahid Mahmud, Hamid Latif Siddiqui

**Affiliations:** a Applied Chemistry Research Centre, PCSIR Laboratories Complex, Lahore 54600, Pakistan; b Chemistry Department, Loughborough University, Loughborough LE11 3TU, England; c Institute of Chemistry, University of The Punjab, Lahore 54590, Pakistan

## Abstract

In the title compound, C_8_H_8_N_4_O_5_, the nitro groups *ortho* and *para* to the hydrazone group are twisted by 10.0 (2) and 3.6 (2)°, respectively, relative to the aromatic ring. The structure exhibits an intra­molecular N—H⋯O hydrogen bond between the hydrazide and *ortho*-nitro groups. There is a strong inter­molecular C=O⋯H—N hydrogen bond, giving rise to chains, and weaker ONO⋯NO_2_ [2.944 (2) Å] and C—H⋯O—N inter­actions linking the mol­ecules into a three-dimensional network.

## Related literature

For related literature, see: Domiano *et al.* (1984 ▶); Guo (2007 ▶); Li *et al.* (1988 ▶); Rudnicka & Osmialowska (1979 ▶); Sakamoto *et al.* (1993 ▶); Siddiqui *et al.* (2007 ▶); Zia-ur-Rehman *et al.* (2005 ▶, 2006 ▶). 
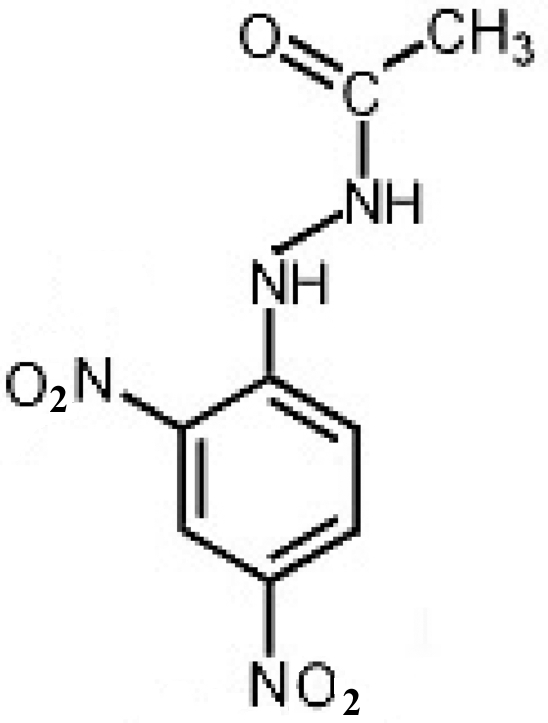


## Experimental

### 

#### Crystal data

C_8_H_8_N_4_O_5_
*M* *_r_* = 240.18
Orthorhombic, 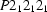

*a* = 4.8585 (4) Å
*b* = 10.7703 (8) Å
*c* = 19.1059 (14) Å
*V* = 999.76 (13) Å^3^
*Z* = 4
Mo *K*α radiation
μ = 0.14 mm^−1^
*T* = 150 (2) K
0.57 × 0.09 × 0.06 mm

#### Data collection

Bruker APEXII CCD diffractometer
Absorption correction: multi-scan (*SADABS*; Sheldrick, 2007 ▶) *T*_min_ = 0.927, *T*_max_ = 0.992
11843 measured reflections
1794 independent reflections
1616 reflections with *I* > 2σ(*I*)
*R*_int_ = 0.031

#### Refinement

*R*[*F* ^2^ > 2σ(*F* ^2^)] = 0.030
*wR*(*F* ^2^) = 0.078
*S* = 1.03
1794 reflections
161 parameters
H atoms treated by a mixture of independent and constrained refinement
Δρ_max_ = 0.27 e Å^−3^
Δρ_min_ = −0.17 e Å^−3^

### 

Data collection: *APEX2* (Bruker, 2006 ▶); cell refinement: *SAINT* (Bruker, 2006 ▶); data reduction: *SAINT*; program(s) used to solve structure: *SHELXS97* (Sheldrick, 2008 ▶); program(s) used to refine structure: *SHELXL97* (Sheldrick, 2008 ▶); molecular graphics: *SHELXTL* (Sheldrick, 2008 ▶); software used to prepare material for publication: *SHELXTL* and local programs.

## Supplementary Material

Crystal structure: contains datablocks I, global. DOI: 10.1107/S1600536808019685/bt2733sup1.cif

Structure factors: contains datablocks I. DOI: 10.1107/S1600536808019685/bt2733Isup2.hkl

Additional supplementary materials:  crystallographic information; 3D view; checkCIF report

## Figures and Tables

**Table 1:** Hydrogen-bond geometry (Å, °)

*D*—H⋯*A*	*D*—H	H⋯*A*	*D*⋯*A*	*D*—H⋯*A*
N3—H3⋯O2	0.83 (2)	2.001 (18)	2.5942 (16)	127.9 (16)
N4—H4⋯O5^i^	0.85 (2)	1.95 (2)	2.7748 (16)	164.0 (17)
C5—H5⋯O4^ii^	0.95	2.44	3.249 (2)	143
C8—H8*A*⋯O2^iii^	0.98	2.58	3.269 (2)	128
C8—H8*C*⋯O3^iv^	0.98	2.57	3.527 (2)	165

Symmetry code: (i) 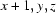; (ii) 
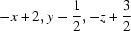
; (iii) 
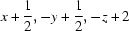
; (iv) 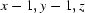.

**Fig. 1.**
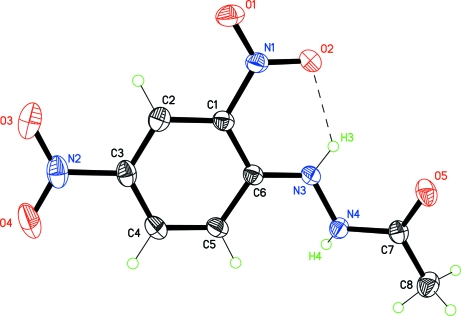
The asymmetric unit of the title compound showing the intramolecular hydrogen bond. Displacement ellipsoids are drawn at the 50% probability level.

**Fig. 2.**
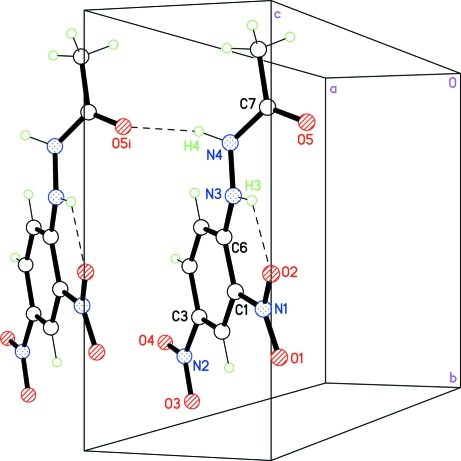
Perspective view of molecules linked via intermolecular N—H···O=C hydrogen bonds parallel to a.

**Fig. 3.**
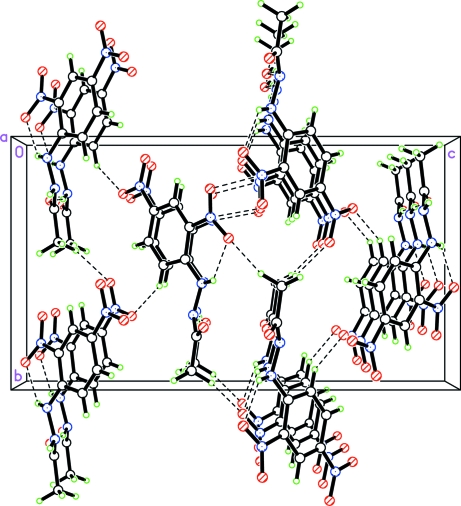
Perspective view of the three-dimensional crystal packing showing hydrogen-bonds and other intermolecular interactions (dashed lines). H atoms not involved in hydrogen bonding have been omitted for clarity.

## supplementary crystallographic information

### Comment

The chemistry of hydrazones has been intensely investigated in recent years due to their excellent coordinating capability (Domiano *et al.*, 1984) and pharmacological activities (Li *et al.*, 1988). These compounds are also being used as precursors for the efficient synthesis of various condensed heterocycles in organic chemistry (Rudnicka & Osmialowska, 1979) and as highly selective metal scavengers (Sakamoto *et al.*, 1993) in analytical chemistry. In continuation of our ongoing work on the synthesis of various heterocyclic compounds (Zia-ur-Rehman *et al.*, 2005, 2006; Siddiqui *et al.*, 2007), the title compound, (I), was synthesized by reacting 2,4-dinitrophenylhydrazine with acetic anhydride.

Most of the bond lengths and angles in (I) are similar to those in related molecules (Guo, 2007). The nitro groups *ortho* and *para* to the hydrazone group are twisted out of this plane by 10.0 (2) and 3.6 (2)°, respectively. The larger twist of the *ortho*-nitro group arises due to the desire to form an intramolecular hydrogen bond which results in a six-membered ring (Fig. 1 and Table 1). Each molecule also forms an intermolecular N—H···O═C hydrogen bond giving rise to stacks of molecules parallel to *a* (Fig. 2). The hydrogen-bonded chains of (I) are further linked together into a three-dimensional network (Fig. 3) *via* weaker C—H···O—N interactions involving the nitro groups and methyl and aryl H atoms (range 2.4–2.6Å) along with some weak ONO···NO_2_ interactions [O1···N1^i^ = 2.944 (2)Å; symmetry code: (i) -0.5+x,  1.5-y, 2-z].

### Experimental

A mixture of 2,4-dinitrophenylhydrazine (1.981 g; 10.0 mmoles) and acetic anhydride (5.0 ml) was stirred for a period of six hours at room temperature. Then, this mixture was poured into ice cooled water and neutralized with 10% sodium bicarbonate solution. The precipitated solids were collected by filtration, washed and dried. Crystals suitable for X-ray crystallography were grown by slow evaporation of solution of the title compound in a mixture of ethanol and water (90:10); m.p. 471 K; yield: 82%.

### Refinement

1255 Friedel pairs were merged. H atoms bound to C were placed in geometric positions (C—H distance = 0.95 Å for aryl-H; 0.98 Å for methyl-H) using a riding model. H atoms on N had coordinates freely refined. *U*_iso_ values were set to 1.2*U*_eq_ of the carrier atom (1.5*U*_eq_ for methyl-H).

### Crystal data

**Table:**

C_8_H_8_N_4_O_5_	*F*_000_ = 496
*M**_r_* = 240.18	*D*_x_ = 1.596 Mg m^−^^3^
Orthorhombic, *P*2_1_2_1_2_1_	Mo *K*α radiation λ = 0.71073 Å
Hall symbol: P 2ac 2ab	Cell parameters from 3491 reflections
*a* = 4.8585 (4) Å	θ = 2.9–27.8º
*b* = 10.7703 (8) Å	µ = 0.14 mm^−^^1^
*c* = 19.1059 (14) Å	*T* = 150 (2) K
*V* = 999.76 (13) Å^3^	Lath, orange
*Z* = 4	0.57 × 0.09 × 0.06 mm

### Data collection

**Table:**

Bruker APEXII CCD diffractometer	1794 independent reflections
Radiation source: fine-focus sealed tube	1616 reflections with *I* > 2σ(*I*)
Monochromator: graphite	*R*_int_ = 0.031
*T* = 150(2) K	θ_max_ = 30.6º
ω rotation with narrow frames scans	θ_min_ = 2.1º
Absorption correction: multi-scan(SADABS; Sheldrick, 2007)	*h* = −6→6
*T*_min_ = 0.927, *T*_max_ = 0.992	*k* = −15→15
11843 measured reflections	*l* = −27→27

### Refinement

**Table:**

Refinement on *F*^2^	Secondary atom site location: all non-H atoms found by direct methods
Least-squares matrix: full	Hydrogen site location: geom except NH coords freely refined
*R*[*F*^2^ > 2σ(*F*^2^)] = 0.030	H atoms treated by a mixture of independent and constrained refinement
*wR*(*F*^2^) = 0.078	*w* = 1/[σ^2^(*F*_o_^2^) + (0.0462*P*)^2^ + 0.078*P*] where *P* = (*F*_o_^2^ + 2*F*_c_^2^)/3
*S* = 1.03	(Δ/σ)_max_ < 0.001
1794 reflections	Δρ_max_ = 0.27 e Å^−^^3^
161 parameters	Δρ_min_ = −0.17 e Å^−^^3^
Primary atom site location: structure-invariant direct methods	Extinction correction: none

### Special details

**Table:**

Geometry. All e.s.d.'s (except the e.s.d. in the dihedral angle between two l.s. planes) are estimated using the full covariance matrix. The cell e.s.d.'s are taken into account individually in the estimation of e.s.d.'s in distances, angles and torsion angles; correlations between e.s.d.'s in cell parameters are only used when they are defined by crystal symmetry. An approximate (isotropic) treatment of cell e.s.d.'s is used for estimating e.s.d.'s involving l.s. planes.
Refinement. Refinement of *F*^2^ against ALL reflections. The weighted *R*-factor *wR* and goodness of fit *S* are based on *F*^2^, conventional *R*-factors *R* are based on *F*, with *F* set to zero for negative *F*^2^. The threshold expression of *F*^2^ > σ(*F*^2^) is used only for calculating *R*-factors(gt) *etc*. and is not relevant to the choice of reflections for refinement. *R*-factors based on *F*^2^ are statistically about twice as large as those based on *F*, and *R*- factors based on ALL data will be even larger. 1255 Friedel pairs. Friedels merged.

### Fractional atomic coordinates and isotropic or equivalent isotropic displacement parameters (Å^2^)

**Table:**

	*x*	*y*	*z*	*U*_iso_*/*U*_eq_	
C1	0.3396 (3)	0.64098 (13)	0.89834 (7)	0.0206 (3)	
N1	0.1186 (2)	0.67553 (11)	0.94555 (6)	0.0239 (2)	
O1	0.0352 (2)	0.78314 (10)	0.94499 (6)	0.0298 (2)	
O2	0.0219 (2)	0.59512 (11)	0.98457 (6)	0.0349 (3)	
C2	0.4679 (3)	0.73783 (13)	0.86286 (7)	0.0234 (3)	
H2	0.4153	0.8215	0.8710	0.028*	
C3	0.6722 (3)	0.70968 (14)	0.81593 (7)	0.0245 (3)	
N2	0.8161 (3)	0.81093 (13)	0.78132 (7)	0.0323 (3)	
O3	0.7483 (3)	0.91873 (12)	0.79517 (7)	0.0428 (3)	
O4	1.0012 (3)	0.78354 (13)	0.73998 (7)	0.0462 (3)	
C4	0.7508 (3)	0.58725 (15)	0.80240 (7)	0.0253 (3)	
H4A	0.8903	0.5700	0.7689	0.030*	
C5	0.6255 (3)	0.49242 (13)	0.83774 (7)	0.0231 (3)	
H5	0.6791	0.4093	0.8283	0.028*	
C6	0.4174 (3)	0.51517 (13)	0.88817 (7)	0.0200 (3)	
N3	0.3089 (3)	0.42103 (11)	0.92557 (7)	0.0243 (3)	
H3	0.172 (4)	0.4329 (16)	0.9508 (10)	0.029*	
N4	0.3548 (3)	0.29805 (11)	0.90659 (7)	0.0222 (2)	
H4	0.518 (4)	0.2710 (16)	0.9126 (10)	0.027*	
C7	0.1401 (3)	0.21961 (13)	0.91490 (7)	0.0222 (3)	
O5	−0.0894 (2)	0.25786 (10)	0.93114 (6)	0.0291 (2)	
C8	0.2055 (4)	0.08501 (14)	0.90370 (9)	0.0310 (3)	
H8A	0.1747	0.0392	0.9473	0.047*	
H8B	0.3984	0.0764	0.8895	0.047*	
H8C	0.0861	0.0515	0.8669	0.047*	

### Atomic displacement parameters (Å^2^)

**Table:**

	*U*^11^	*U*^22^	*U*^33^	*U*^12^	*U*^13^	*U*^23^
C1	0.0161 (6)	0.0247 (6)	0.0211 (6)	−0.0007 (5)	−0.0003 (5)	−0.0036 (5)
N1	0.0187 (5)	0.0254 (5)	0.0276 (6)	−0.0018 (5)	0.0007 (5)	−0.0083 (5)
O1	0.0243 (5)	0.0272 (5)	0.0380 (6)	0.0046 (4)	−0.0021 (5)	−0.0092 (4)
O2	0.0334 (6)	0.0304 (6)	0.0408 (6)	−0.0044 (5)	0.0178 (5)	−0.0053 (5)
C2	0.0218 (6)	0.0241 (6)	0.0242 (6)	−0.0029 (5)	−0.0050 (5)	0.0001 (5)
C3	0.0229 (6)	0.0303 (7)	0.0203 (6)	−0.0070 (6)	−0.0027 (5)	0.0046 (5)
N2	0.0323 (7)	0.0384 (7)	0.0262 (6)	−0.0125 (6)	−0.0069 (6)	0.0095 (5)
O3	0.0475 (8)	0.0320 (6)	0.0489 (7)	−0.0124 (6)	−0.0052 (6)	0.0114 (5)
O4	0.0430 (7)	0.0581 (8)	0.0374 (6)	−0.0177 (7)	0.0111 (6)	0.0105 (6)
C4	0.0203 (7)	0.0352 (7)	0.0203 (6)	−0.0025 (6)	0.0020 (5)	0.0001 (6)
C5	0.0205 (6)	0.0267 (6)	0.0221 (6)	0.0012 (5)	0.0029 (5)	−0.0024 (5)
C6	0.0161 (6)	0.0238 (6)	0.0200 (6)	−0.0007 (5)	−0.0006 (5)	−0.0014 (5)
N3	0.0210 (6)	0.0217 (5)	0.0301 (6)	0.0006 (5)	0.0084 (5)	−0.0019 (5)
N4	0.0154 (5)	0.0200 (5)	0.0312 (6)	0.0011 (4)	0.0006 (5)	−0.0004 (5)
C7	0.0182 (6)	0.0256 (6)	0.0228 (6)	−0.0011 (5)	−0.0022 (5)	0.0029 (5)
O5	0.0159 (5)	0.0339 (6)	0.0375 (6)	−0.0002 (4)	0.0012 (4)	0.0046 (5)
C8	0.0308 (8)	0.0235 (6)	0.0389 (8)	−0.0011 (6)	−0.0008 (7)	0.0019 (6)

### Geometric parameters (Å, °)

**Table:**

C1—C2	1.3915 (19)	C5—C6	1.4178 (18)
C1—C6	1.4202 (19)	C5—H5	0.9500
C1—N1	1.4508 (18)	C6—N3	1.3477 (18)
N1—O1	1.2278 (16)	N3—N4	1.3913 (17)
N1—O2	1.2354 (16)	N3—H3	0.83 (2)
C2—C3	1.371 (2)	N4—C7	1.3519 (18)
C2—H2	0.9500	N4—H4	0.85 (2)
C3—C4	1.397 (2)	C7—O5	1.2282 (17)
C3—N2	1.4543 (19)	C7—C8	1.500 (2)
N2—O4	1.233 (2)	C8—H8A	0.9800
N2—O3	1.2355 (19)	C8—H8B	0.9800
C4—C5	1.367 (2)	C8—H8C	0.9800
C4—H4A	0.9500		
			
C2—C1—C6	121.96 (13)	C6—C5—H5	119.2
C2—C1—N1	116.25 (12)	N3—C6—C5	120.61 (13)
C6—C1—N1	121.79 (12)	N3—C6—C1	122.76 (12)
O1—N1—O2	122.79 (12)	C5—C6—C1	116.59 (12)
O1—N1—C1	118.74 (12)	C6—N3—N4	121.01 (12)
O2—N1—C1	118.47 (12)	C6—N3—H3	120.3 (12)
C3—C2—C1	118.50 (13)	N4—N3—H3	115.2 (12)
C3—C2—H2	120.8	C7—N4—N3	116.14 (12)
C1—C2—H2	120.8	C7—N4—H4	119.2 (12)
C2—C3—C4	121.83 (13)	N3—N4—H4	116.0 (13)
C2—C3—N2	118.65 (14)	O5—C7—N4	121.37 (13)
C4—C3—N2	119.49 (13)	O5—C7—C8	123.55 (14)
O4—N2—O3	123.81 (14)	N4—C7—C8	115.07 (13)
O4—N2—C3	117.56 (14)	C7—C8—H8A	109.5
O3—N2—C3	118.63 (15)	C7—C8—H8B	109.5
C5—C4—C3	119.47 (13)	H8A—C8—H8B	109.5
C5—C4—H4A	120.3	C7—C8—H8C	109.5
C3—C4—H4A	120.3	H8A—C8—H8C	109.5
C4—C5—C6	121.60 (13)	H8B—C8—H8C	109.5
C4—C5—H5	119.2		
			
C2—C1—N1—O1	9.02 (18)	N2—C3—C4—C5	176.53 (13)
C6—C1—N1—O1	−170.04 (13)	C3—C4—C5—C6	−0.2 (2)
C2—C1—N1—O2	−171.10 (12)	C4—C5—C6—N3	−175.76 (14)
C6—C1—N1—O2	9.84 (19)	C4—C5—C6—C1	2.1 (2)
C6—C1—C2—C3	1.1 (2)	C2—C1—C6—N3	175.25 (13)
N1—C1—C2—C3	−177.94 (12)	N1—C1—C6—N3	−5.7 (2)
C1—C2—C3—C4	0.9 (2)	C2—C1—C6—C5	−2.53 (19)
C1—C2—C3—N2	−177.00 (13)	N1—C1—C6—C5	176.47 (12)
C2—C3—N2—O4	179.13 (13)	C5—C6—N3—N4	−13.1 (2)
C4—C3—N2—O4	1.2 (2)	C1—C6—N3—N4	169.18 (13)
C2—C3—N2—O3	−0.3 (2)	C6—N3—N4—C7	−142.12 (14)
C4—C3—N2—O3	−178.27 (14)	N3—N4—C7—O5	7.5 (2)
C2—C3—C4—C5	−1.3 (2)	N3—N4—C7—C8	−171.84 (12)

### Hydrogen-bond geometry (Å, °)

**Table:**

*D*—H···*A*	*D*—H	H···*A*	*D*···*A*	*D*—H···*A*
N3—H3···O2	0.83 (2)	2.001 (18)	2.5942 (16)	127.9 (16)
N4—H4···O5^i^	0.85 (2)	1.95 (2)	2.7748 (16)	164.0 (17)
C5—H5···O4^ii^	0.95	2.44	3.249 (2)	143
C8—H8A···O2^iii^	0.98	2.58	3.269 (2)	128
C8—H8C···O3^iv^	0.98	2.57	3.527 (2)	165

Symmetry codes: (i) *x*+1, *y*, *z*; (ii) −*x*+2, *y*−1/2, −*z*+3/2; (iii) *x*+1/2, −*y*+1/2, −*z*+2; (iv) *x*−1, *y*−1, *z*.
